# Supplementary material for: Psychiatric symptomatology in skin-restricted lupus patients without axis I psychiatric disorders: A post-hoc analysis
Source: PLoS One. 2023 Mar 1;18(3):e0282079. doi: 10.1371/journal.pone.0282079 (PMC9977055; doi:10.1371/journal.pone.0282079)
Supplement: S1 Fig — Patients with high MADRS scores (extreme values) were not the same across visits. (DOCX) [file pone.0282079.s001.docx]

**S1 Fig. Patients’ MADRS and HAMA scores during the follow-up**


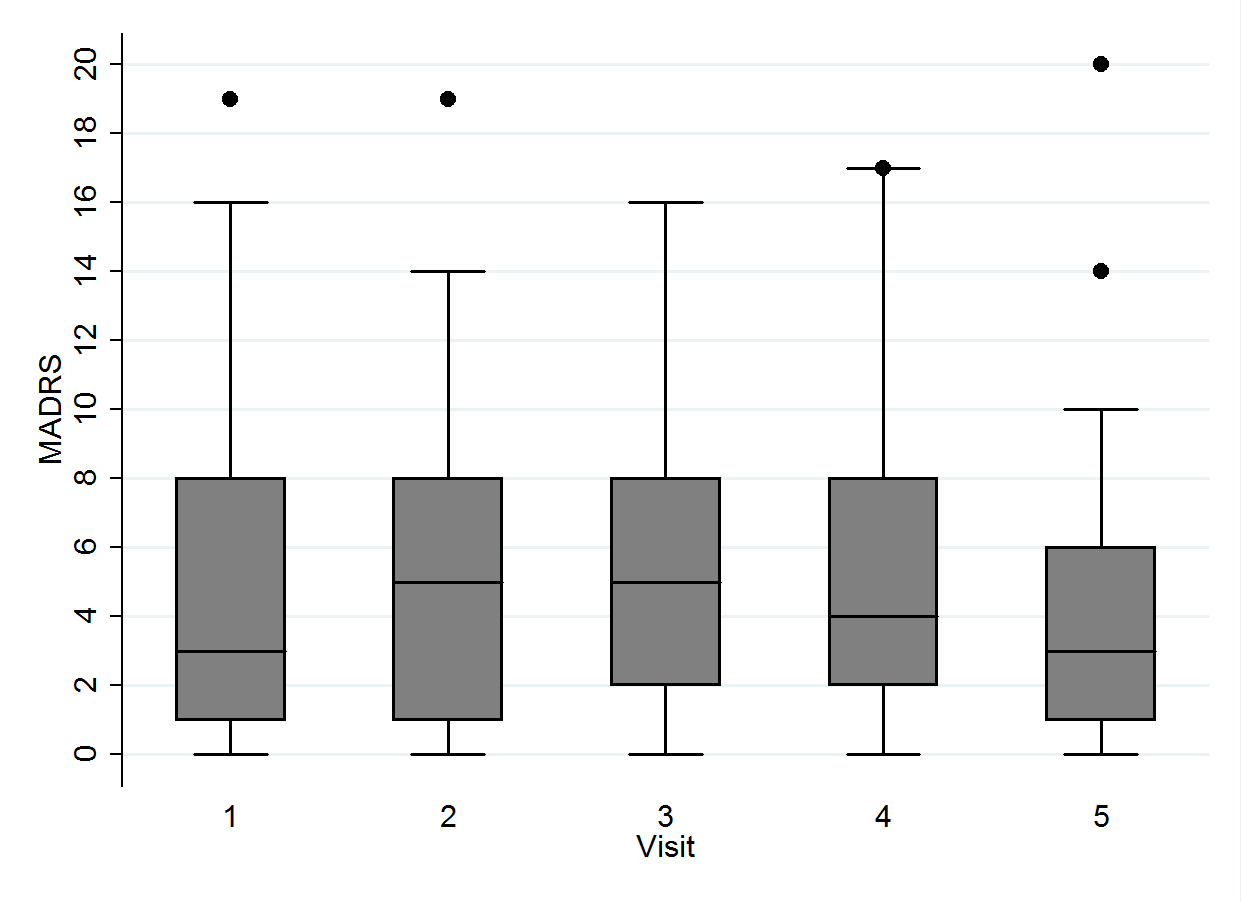


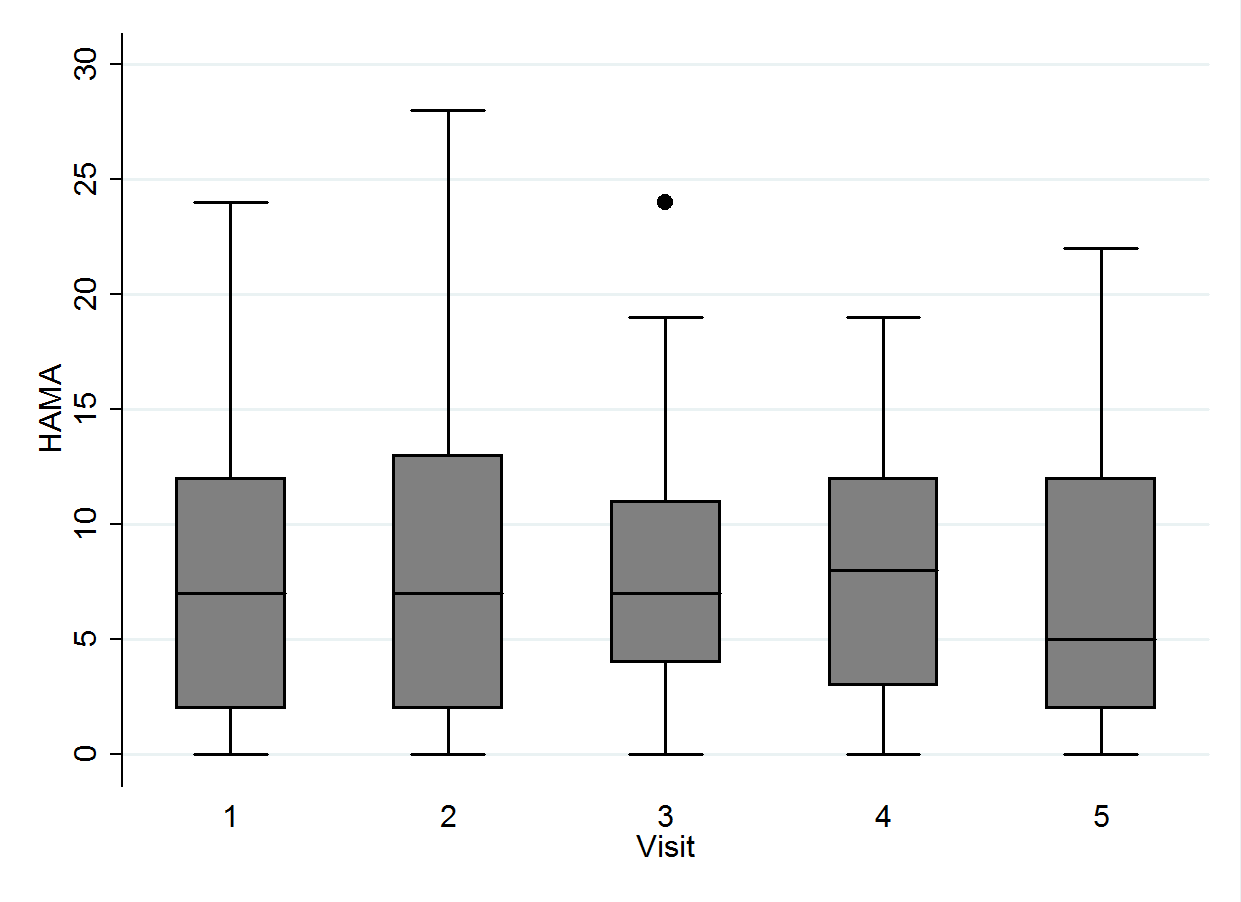


Patients with high MADRS scores (extreme values) were not the same across visits.
