# Supplementary material for: Psychiatric symptomatology in skin-restricted lupus patients without axis I psychiatric disorders: A post-hoc analysis
Source: PLoS One. 2023 Mar 1;18(3):e0282079. doi: 10.1371/journal.pone.0282079 (PMC9977055; doi:10.1371/journal.pone.0282079)
Supplement: S1 Table — Symptoms scores are presented as median [interquartile range]. † For age, spearman rho coefficients are presented. *Significantly different, p<0.05; ** p<0.01 and *** p<0.001. (DOCX) [file pone.0282079.s002.docx]

**S1 Table. Factors affecting symptom scores in participants.**

|  | **Depressive** | **Neurovegetative** | **Psychic anxiety** | **Somatic** |
| --- | --- | --- | --- | --- |
| Age ^†^ | -0.05 | 0.05 | -0.08 | 0.11 |
| Female | 0 [0 - 1] | 0 [0 - 2] | 1 [0 - 3] | 1 [0 - 3] |
| Male | 0 [0 - 0] | 0 [0 - 1] | 1 [0 - 2] | 1 [0 - 1] |
| Smokers | 0 [0 - 1] | 0 [0 - 2] | 2 [0 - 3] | 1 [0 - 3] |
| Non-smokers | 0 [0 - 2] | 0 [0 - 2] | 1 [0 - 2] | 1 [0 - 2] |
| Medical comorbidities | 0 [0 - 1.5] | 0 [0 - 2] | 1 [0 - 3] | 1 [0 - 4] |
| No medical comorbidity | 0 [0 - 1] | 0 [0 - 2] | 1 [0 - 2] | 1 [0 - 2] |
| *Cardiovascular* | *0 [0 - 1]* | *0 [0 - 0.5]* | *1.5 [0 - 3.5]* | *1 [0 - 4]* |
| *No cardiovascular* | *0 [0 - 1]* | *0 [0 - 2]* | *1 [0 - 3]* | *1 [0 - 3]* |
| *Digestive system* | *0 [0 - 0]* | *0 [0 - 2]* | *1 [0 - 3]* | *1 [0 - 2]* |
| *No digestive system* | *0 [0 - 2]* | *0 [0 - 2]* | *1 [0 - 3]* | *1 [0 - 3]* |
| *Endocrine, nutritional or metabolic* | *0 [0 - 0]* | *0 [0 - 1]* | *1 [1 - 2]* | *0 [0 - 7]* |
| *No endocrine…* | *0 [0 - 2]* | *0 [0 - 2]* | *1 [0 - 3]* | *1 [0 - 3]* |
| *Genitourinary system* | *1 [0 - 2]* | *3 [0 - 6]* | *2 [0 - 5]* | *4 [1 - 5]* |
| *No genitourinary system* | *0 [0 - 1]* | *0 [0 - 2]* | *1 [0 - 3]* | *1 [0 - 3]* |
| *Infection* | *2 [0 - 8]* | *0 [0 - 6]* | *3 [1 - 6]* | *2 [1 - 8]* |
| *No infection* | *0 [0 - 1]* | *0 [0 - 2]* | *1 [0 - 3]* | *1 [0 - 3]* |
| *Osteo-articular system, muscles and connective tissue* | *0 [0 - 0]* | *0 [0 - 2]* | *1.5 [0 - 3]* | *1 [0 - 2]* |
| *No osteo-articular system…* | *0 [0 - 1.5]* | *0 [0 - 1]* | *1 [0 - 3]* | *1 [0 - 3]* |
| *Tumor* | *0 [0 - 2]* | *0 [0 - 1]* | *0.5 [0 - 5]* | *1.5 [0 - 7]* |
| *No tumor* | *0 [0 - 1]* | *0 [0 - 2]* | *1 [0 - 3]* | *1 [0 - 3]* |
| Current psychotropic treatment | 0 [0 - 2] | 0 [0 - 4] | 4 [2 - 7] ** | 6 [1 - 8] * |
| No current psychotropic treatment | 0 [0 - 1] | 0 [0 - 2] | 1 [0 - 2] | 1 [0 - 2] |
| *Current antidepressant* | *0 [0 - 0]* | *0 [0 - 0]* | *4 [4 - 7] *** | *7 [6 - 8] *** |
| *No antidepressant* | *0 [0 - 1]* | *0 [0 - 2]* | *1 [0 - 2]* | *1 [0 - 2]* |
| *Current anxiolytic* | *0 [0 - 0]* | *0 [0 - 0]* | *4 [4 - 9] ** | *8 [1 - 8]* |
| *No anxiolytic* | *0 [0 - 2]* | *0 [0 - 2]* | *1 [0 - 3]* | *1 [0 - 3]* |
| *Current hypnotic* | *1 [0 - 2]* | *2 [0 - 4]* | *1.5 [1 - 2]* | *3 [0 - 6]* |
| *No hypnotic* | *0 [0 - 1]* | *0 [0 - 2]* | *1 [0 - 3]* | *1 [0 - 3]* |
| Current personality disorder | 2 [0 - 4] *** | 0.5 [0 - 2] | 2.5 [1 - 5] ** | 1 [0 - 6] |
| No current personality disorder | 0 [0 - 0] | 0 [0 -2] | 1 [0 - 2] | 1 [0 - 2] |
| Past psychiatric disorder | 0 [0 - 2] | 0 [0 - 2] | 2 [0 - 3] | 1 [0 - 4] |
| No past psychiatric disorder | 0 [0 - 0] | 0 [0 - 2] | 1 [0 - 2] | 1 [0 - 3] |
| Past psychotropic treatment | 0 [0 - 2] | 0 [0 - 2] | 2 [1 - 4] ** | 2 [1 - 6] ** |
| No past psychotropic treatment | 0 [0 - 1] | 0 [0 - 2] | 1 [0 - 2] | 1 [0 - 2] |
| *Past antidepressant* | *0 [0 - 1]* | *0 [0 - 1]* | *2 [1 - 4]* | *1 [0 - 8]* |
| *No antidepressant* | *0 [0 - 2]* | *0 [0 - 2]* | *1 [0 - 3]* | *1 [0 - 2]* |
| *Past anxiolytic* | *0 [0 - 2]* | *0 [0 - 2]* | *2 [0 - 5]* | *3 [1 - 7] *** |
| *No anxiolytic* | *0 [0 - 1]* | *0 [0 - 2]* | *1 [0 - 2]* | *1 [0 - 2]* |
| *Past hypnotic* | *0.5 [0 - 1.5]* | *0 [0 - 2]* | *3 [2 - 3]* | *1.5 [0.5 - 5]* |
| *No hypnotic* | *0 [0 - 1]* | *0 [0 - 2]* | *1 [0 - 2]* | *1 [0 - 3]* |

Symptoms scores are presented as median [interquartile range].

^†^ For age, spearman rho coefficients are presented.

*Significantly different, p<0.05; ** p<0.01 and *** p<0.001.
