# Supplementary material for: Psychiatric symptomatology in skin-restricted lupus patients without axis I psychiatric disorders: A post-hoc analysis
Source: PLoS One. 2023 Mar 1;18(3):e0282079. doi: 10.1371/journal.pone.0282079 (PMC9977055; doi:10.1371/journal.pone.0282079)
Supplement: S3 Table — Results are presented as N (%), mean ± SD or median [inter quartile range]. Bold values indicate significant results with a p-value < 0.05. † 3 missing values. †† Value for 21 patients. ††† A total of 23 areas were considered: face, neck, collar, ears, scalp, anterior and posterior sides of the chest, abdomen, lumbar area, left and right arms, left and right forearms, palmar sides of left and right hands, dorsal sides of left and right hands, left and right thighs, left and right legs, dorsal sides of left and right feet. (DOCX) [file pone.0282079.s004.docx]

**S3 Table. Factors associated with occurrence of psychiatric disorders in skin-restricted lupus patients.**

|  | Patients with psychiatric disorders  N = 9 | Patients without psychiatric disorders  N = 24 | pValue |
| --- | --- | --- | --- |
| Sex (Female) | 8 (89) | 19 (79) | 1.000 |
| Age | 46.2 ± 17.4 | 44.6 ± 12.8 | 0.824 |
| Smokers | 5 (63) | 15 (63) | 1.000 |
| Lupus type |  |  |  |
| Chronic cutaneous | 5 (56) | 19 (79) | 0.212 |
| Subacute | 4 (44) | 5 (21) |  |
| Lupus duration, years | 4.3 [2.9 - 11.1] | 6.3 [2.5 - 10.8] | 0.857 |
| Largest lesion size, cm^2^ | 2.2 [0.8 - 4.8] | 1.0 [0.2 - 5.8] | 0.679 |
| Number of lupus lesions | 2 [1 - 3.5] | 2 [1 - 7] | 0.913 |
| Number of affected areas | 1 [1 - 1] | 1 [1 - 3] | 0.431 |
| Pruritus or burning sensations | 2 (25) | 13 (54) | 0.229 |
| CLASI ^††^ |  |  |  |
| Activity | 2.5 [1 - 4] | 3 [1 - 4] | 0.913 |
| Damage | 0 [0 - 3] | 1 [0 - 2] | 0.717 |
| Lesions on visible areas ^†††^ | 6 (75) | 16 (67) | 1.000 |
| Current lupus treatment | 8 (89) | 21 (88) | 1.000 |
| Synthetic antimalarials | 3 (33) | 10 (42) | 1.000 |
| Thalidomide | 4 (44) | 6 (25) | 0.400 |
| Topical steroids | 1 (11) | 6 (25) | 0.642 |
| Current psychotropic treatment | 2 (22) | 4 (17) | 1.000 |
| Antidepressant | 2 (22) | 3 (13) | 1.000 |
| Anxiolytic | 1 (11) | 2 (8) | 1.000 |
| Hypnotic | 0 (0) | 1 (4) | 1.000 |
| Psychiatrist consultation | 0 (0) | 0 (0) | - |
| Current personality disorder ^†^ | 4 (44) | 5 (24) | 0.389 |
| Past psychiatric disorder | 4 (44) | 12 (50) | 1.000 |
| Past psychotropic treatment | **8 (89)** | **8 (33)** | **0.007** |
| Antidepressant | 4 (44) | 5 (21) | 0.212 |
| Anxiolytic | 4 (44) | 8 (33) | 0.690 |
| Hypnotic | **4 (44)** | **1 (4)** | **0.013** |
| Past psychiatrist consultation | 2 (22) | 3 (13) | 0.597 |
| MADRS Total | 6 [2 - 11] | 2 [0 - 6.5] | 0.082 |
| Depressive symptoms | 1 [0 - 4] | 0 [0 - 1.5] | 0.303 |
| Neurovegetative symptoms | 3 [0 - 5] | 0 [0 - 2] | 0.115 |
| HAMA Total | **18 [6 - 21]** | **4.5 [1 - 10]** | **0.012** |
| Psychic anxiety symptoms | **4 [3 - 6]** | **1.5 [0 - 3.5]** | **0.008** |
| Somatic symptoms | 7 [0 - 8] | 1.5 [0 - 4.5] | 0.140 |

Results are presented as N (%), mean ± SD or median [inter quartile range].

Bold values indicate significant results with a p-value < 0.05.

^†^ 3 missing values

**^††^** Value for 21 patients

**^†††^** A total of 23 areas were considered: face, neck, collar, ears, scalp, anterior and posterior sides of the chest, abdomen, lumbar area, left and right arms, left and right forearms, palmar sides of left and right hands, dorsal sides of left and right hands, left and right thighs, left and right legs, dorsal sides of left and right feet.
