# Supplementary material for: Psychiatric symptomatology in skin-restricted lupus patients without axis I psychiatric disorders: A post-hoc analysis
Source: PLoS One. 2023 Mar 1;18(3):e0282079. doi: 10.1371/journal.pone.0282079 (PMC9977055; doi:10.1371/journal.pone.0282079)
Supplement: Attachment — Submitted filename: Response to Reviewers.docx [file pone.0282079.s005.docx]

**Response to the academic editor**

1. Please ensure that your manuscript meets PLOS ONE's style requirements, including those for file naming.

Style requirement has been checked and changed when required.

2. We note that you have indicated that data from this study are available upon request. PLOS only allows data to be available upon request if there are legal or ethical restrictions on sharing data publicly. For more information on unacceptable data access restrictions, please see <http://journals.plos.org/plosone/s/data-availability#loc-unacceptable-data-access-restrictions>.

In your revised cover letter, please address the following prompts:

a) If there are ethical or legal restrictions on sharing a de-identified data set, please explain them in detail (e.g., data contain potentially sensitive information, data are owned by a third-party organization, etc.) and who has imposed them (e.g., an ethics committee). Please also provide contact information for a data access committee, ethics committee, or other institutional body to which data requests may be sent.

b) If there are no restrictions, please upload the minimal anonymized data set necessary to replicate your study findings as either Supporting Information files or to a stable, public repository and provide us with the relevant URLs, DOIs, or accession numbers. For a list of acceptable repositories, please see <http://journals.plos.org/plosone/s/data-availability#loc-recommended-repositories>.

All the data are now available at Mendeley:

Rondepierre, Fabien; Tauveron-Jalenques, Urbain; Valette, Solène; Mulliez, Aurélien; D'Incan, Michel; Lauron, Sophie; Jalenques, Isabelle (2022), “Psychiatric symptomatology in skin-restricted lupus patients without psychiatric disorders: a post-hoc analysis”, Mendeley Data, V1, doi: 10.17632/bdxf94z5xw.1

While revising your submission, please upload your figure files to the Preflight Analysis and Conversion Engine (PACE) digital diagnostic tool, <https://pacev2.apexcovantage.com/>. PACE helps ensure that figures meet PLOS requirements.

All our figures have been checked with PACE digital diagnostic tool and they meet PLOS requirements.

**Response to reviewers**

Reviewer #1: Thank you for the opportunity to review this manuscript. Please see below for my comments, which I hope prove useful.

Introduction

1.Suggest making it clear that the aim of the study is to investigate the relationship between depressive and anxiety symptoms in patients with SRL without PREVALENT psychiatric disorders. I understand that there might be participants included in this analysis who have a history of depression or an anxiety disorder
We don't usually use "prevalent psychiatric disorders", do you mean “current psychiatric disorders”?

If this is the case, we agree that it is important to specify it. This has been added at the end of the introduction: *“In the present exploratory study, we used longitudinal data to investigate the presence of depressive, psychic anxiety, neurovegetative and somatic symptoms in SRL patients without* ***current*** *psychiatric disorders.”*

Methods

2.Please provide a brief statement about how controls were recruited

We agree that the reference to previous articles is not sufficient (references 3 and 21) and a description of the recruitment of control subjects has been added: “*Data from the studies of the LuPsy cohort were used for this analysis. This cohort comprised 80 consecutive outpatients with chronic SRL (DLE and LT) or SCLE and 160 healthy control subjects* ***without a history of lupus recruited among volunteers from a clinical research centre and workers in public hospitals, the national railway company and administrative personnel of the state education system****.*”

3.Suggest briefly explaining the rationale for using the MADRS and HAMA measures. There measures are a major component of the analyses. Please also describe the rationale for creating the subscales (depressive, neurovegetative, psychic anxiety and somatic symptom scores) from these measures. As a psychiatrist, the terms are a bit confusing, as “depressive” symptoms typically include neurovegetative, anxiety and somatic symptoms. Perhaps a better term would be “depressive cognitions and affect” or something to that effect

The MADRS and HAMA scales are those used in the LuPsy cohort whose data we used again. These scales have the advantage of scanning the various possible symptoms.

As you noticed and as indicated at the end of our discussion in the limit part, symptoms are not always classified in the same manner. These different subscales were previously used in research by numerous authors and we used the same ^[1]^. In addition, this classification is important since we wanted to know which symptom was represented in lupus patients without current psychiatric disorders and which could be associated with the development of psychiatric disorder. Thus we could study if a parallel was possible with the model of cytokine-induced depression (since lupus is an inflammatory disease) with early-onset neurovegetative and somatic symptoms of depression and late-onset psychological symptoms of depression that include mild cognitive alterations and symptoms of depressed mood, sometimes accompanied by anxiety.

4.How were previous psychiatric disorders diagnosed? Also via the MINI or by self-report?

Indeed past psychiatric disorders were also diagnosed via the MINI. This precision has been added in the method part.

5.Please provide the rationale for excluding current Axis 1 psychiatric disorders, but not personality disorders
We agree that there are links between depressive and anxiety disorders and personality disorders. We wanted to study the symptoms present in skin-restricted lupus patients without psychiatric disorders; and also to study whether those patients who present with symptoms have more evolution or not towards a disorder.

We did not exclude personality disorders because a personality disorder is an enduring pattern of inner experience and behavior that deviates markedly from the individual’s culture, is pervasive and inflexible, has an onset in adolescence or early adulthood and is stable over time (DSM).

Our results, presented in table S2, showed that only the depressive symptom score was related to the presence of a current personality disorder in patients. Additionaly the occurrence of a psychiatric disorder during the follow-up period was mostly associated with higher psychic anxiety symptom scores at baseline (Fig 3 and S3 Table); but it was not associated with personality disorders.

6.It is not clear to me exactly how the variables included in the factor analysis (comparing patients with and without incident psychiatric disorders) were selected. This section would benefit from more explanation for transparency

As indicated, the variables considered in this analysis were determined based on the univariate results and clinical relevance among sociodemographic, dermatological and psychiatric data. Thus the HAMA scores and the presence of past psychotropic medication showed difference between patients who developed disorder and those who did not. All other included variables were those that could impact the development of psychiatric disorder on the basis of author’s experience, clinical relevance and bibliographic data such the age and the gender of the patients, the dermatological data (number and size of the lesions, presence of a treatment, type of lupus, presence of pruritus, smoker status) and psychiatric data (history, medication, consultation). As the objective of our work was to investigate the presence and impact of specific symptoms, all HAMA and MADRS symptom scores have been added to the analysis.

Clarifications have been made to this part.

Results

7.The text explaining Figure 2 is very difficult to interpret. Suggest explaining the figure in more detail. I know it represents a factor analysis, but find it hard to understand

We agree that this figure is difficult to interpret and a more detailed explanation has been added: “***The Fig 2A showed the correlation circle of the variables used in the factor analysis on mixed data. The first axis, which represented 24.7% of the variance of the data considered in the analysis, was more linked to the sex, HAMA and MADRS scores, whereas the second axis, with 15.3% of the variance, was more associated with the presence of past psychiatric disorder or lupus or psychiatric medications.*** *The Fig 2B revealed that the patients* ***who developed a psychiatric disorder during the follow-up*** *had a distinctive baseline profile* ***than those who did not develop such disorders. These two profiles were*** *mainly marked by different HAMA scores****, with higher scores for the patients who developed a psychiatric disorder during the follow-up****.*”

8.Boxplots need labels on the X and Y axes and Fig 2 needs a legend

Labels on the X and Y axes have been added on the figures 1 and 3. The figure 2 has been edited for clarity and its legend modified.

Discussion

9.As alluded to above, it is very difficult to determine whether participants may have had past psychiatric diagnoses retrospectively. The method used in this study for diagnosing past psychiatric disorders would help to explain the discrepancy between past psychotropic medication use and past psychiatric disorder in the findings.

The search for past psychiatric disorders was carried out through a psychiatric interview using the MINI, a method which is considered the gold standard. However, we cannot exclude defects in the recall of the disorders, or the psychotropic treatments or the psychiatrist consultations. But the discrepancy between past psychotropic medication use and past psychiatric disorder could above all be explained by the fact that in France psychotropic medications are often prescribed by general practitioners when people complain of psychiatric symptoms even if the necessary criteria are not completely fulfilled to make the diagnosis of psychiatric disorder.

The possibility of recall bias has been added to the discussion on page 16.

10.I wasn’t sure what this paragraph was referring to (page 18):

“Our study hypothesized that the presence of psychiatric symptoms in SRL patients could have been due to the effects of inflammation but post-hoc analysis without inflammation assays failed to establish any such link. However, our study was performed in the context of chronic inflammatory disease with increased cytokine production [1] and no other dermatological factor tested was associated with the different symptoms.”

Where was this analysis done?

We thank you for this remark which leads us to rephrase for more clarity. Our post-hoc analysis hypothesized that the presence of psychiatric symptoms in SRL patients could have been due to the effects of inflammation but the initial cohort study had not included a biological assay thus it was not possible to study such link via a biological analysis.

This sentence has been rewritten for clarity.

1. - Dantzer R, O’Connor JC, Freund GG, Johnson RW, Kelley KW. From inflammation to sickness and depression: when the immune system subjugates the brain. Nat Rev Neurosci. janv 2008;9(1):46‑56.

   - Capuron L, Neurauter G, Musselman DL, Lawson DH, Nemeroff CB, Fuchs D, et al. Interferon-alpha–induced changes in tryptophan metabolism. Biological Psychiatry. nov 2003;54(9):906‑14.

   - Kovacs D, Kovacs P, Eszlari N, Gonda X, Juhasz G. Psychological side effects of immune therapies: symptoms and pathomechanism. Current Opinion in Pharmacology. août 2016;29:97‑103.

   - Anderson G, Berk M, Maes M. Biological phenotypes underpin the physio-somatic symptoms of somatization, depression, and chronic fatigue syndrome. Acta Psychiatr Scand. févr 2014;129(2):83‑97.

   - Majd M, Saunders EFH, Engeland CG. Inflammation and the dimensions of depression: A review. Frontiers in Neuroendocrinology. janv 2020;56:100800.
