# Supplementary material for: Psychiatric symptomatology in skin-restricted lupus patients without axis I psychiatric disorders: A post-hoc analysis
Source: PLoS One. 2023 Mar 1;18(3):e0282079. doi: 10.1371/journal.pone.0282079 (PMC9977055; doi:10.1371/journal.pone.0282079)
Supplement: Attachment — Submitted filename: Response to reviewers.docx [file pone.0282079.s006.docx]

PONE-D-21-34055R1

Psychiatric symptomatology in skin-restricted lupus patients without psychiatric disorders: a post-hoc analysis

PLOS ONE

**Response to reviewers**

**6. Review Comments to the Author**

Reviewer #2: Thank you for the opportunity to review this manuscript.
This paper aims to investigate the subclinical psychiatric symptoms in skin-restricted lupus patients without psychiatric disorders and to study the occurrence of psychiatric disorders during the study follow-up. The study shows that skin-restricted lupus patients experience depression and anxiety symptoms and that those with marked symptoms are at risk of developing psychiatric disorders. The major limitation of the study is that the group of patients had twice as many current personality disorders and twice as much past use of psychotropic medications compared to controls. Those variables are shown in the study to be associated with their subclinical psychiatric symptoms in addition to their cutaneous lupus. The particular contribution of their skin disease to their psychiatric symptoms is unclear.

Please see below for my detailed comments:

I. Title:
• It seems that the term “patients without psychiatric disorders” does not define properly this group of patients who have a high prevalence of personality disorders that is much higher than in the general population. I suggest refining this term.

**In order to be more accurate, we have changed “patients without psychiatric disorders” to “patients without axis I psychiatric disorder” throughout the text.**

II. Abstract:
• Although the authors’ aim is to investigate anxiety and depressive symptoms in skin-restricted lupus patients without psychiatric disorders the patients had a higher prevalence of psychiatric morbidity including current personality disorder and a history of psychotropic medications than controls, which cannot be overlooked. The abstract should emphasize the differences between groups either in the results or the limitations section.

**The differences between lupus patients and controls have been added in the result section of the abstract:** “*None of the participants had any current axis I psychiatric disorders but the patients had personality disorders more frequently and had received more past psychotropic treatments than the controls.*”

• The results should include the multivariate analysis as well that shows that current personality disorder and a history of psychotropic medications were associated with higher MADRS and HAMA scores as well.

**The results of the multivariate analysis have been included in the abstract:** “No dermatological factor tested was associated with these scores. , whereas being a lupus patient was associated with higher neurovegetative and somatic symptoms scores, having a current personality disorder with higher depressive and neurovegetative scores and receiving more past psychotropic treatments with psychic anxiety and somatic symptoms scores.”

• The second aim of the study regarding the occurrence of psychiatric disorders during the study follow-up (as mentioned in the results section of the abstract) should be included in the abstract.

**This second aim has been added to the abstract:** “It was decided, therefore, to investigate the presence of specific symptoms in skin-restricted lupus patients without axis I psychiatric disorders and their impact on the occurrence of axis I psychiatric disorders during the study follow-up.”

III. Introduction:
• The authors note the connection between depression and anxiety and chronic inflammatory diseases and list diseases “such as psoriasis, alopecia areata, atopic dermatitis, systemic lupus erythematosus, inflammatory bowel disease, multiple sclerosis and rheumatoid arthritis” but subsequently bring examples from depression and anxiety observed in obesity and coronary heart disease which are not classical inflammatory diseases. Instead, citing the literature about depression and anxiety in classic chronic inflammatory diseases is reasonable.

**At the end of the introduction, we have added references about depression and anxiety in classic chronic inflammatory diseases.** “Since then, many studies have looked for links between inflammation and depressive symptoms, including in chronic inflammatory diseases [4,6,8,9,19]”^[1]^.

**Regarding obesity, diabetes and coronary heart disease which are not classical inflammatory diseases, but are associated with inflammation we have edited the sentence and started a new paragraph to avoid confusion and improve clarity:** Additionnally, inflammation and depressive symptoms were observed in association in obese men and in diabetic patients [20,21]. In acute coronary heart disease patients with no history of depression, inflammation was associated with somatic symptoms [22]. In the same study, a longitudinal association was also found between inflammation and anxious symptoms [22].”

• There is no explanation and no reference to the statement that: “It would be useful, therefore, to identify specific symptoms associated with chronic inflammatory disease”. Relevant literature about the importance of identifying subclinical depression and anxiety including the usefulness of interventions in preventing progression may be of interest.

**We cited references about the importance of identifying symptoms in the previous sentence:** “These subclinical disorders could be risk factors for progression towards genuine depression and anxiety disorders [13,15]^[2]^”

**We have added a sentence and reference on the effectiveness of symptom management in preventing progression: “**Additionally, interventions in preventing progression may be of interest [16]”**^[3]^.**

IV. Discussion:
• The authors indicate tumor necrosis factor (TNF) antagonist treatment, as a potential treatment for depression in patients with elevated CRP and mention its role in treating dermatologic patients. It is not clear what the authors tried to say. Do they imply the relevance to cutaneous lupus? Although TNF antagonists are indeed used in some chronic inflammatory dermatologic diseases like psoriasis, they have no role in treating cutaneous lupus. This issue should be cleared.
**For clarity, we have removed this passage a little further from the discussion.**

• The discussion should include major limitations including the small number of patients and The basic differences between patients and controls which might confound the results albeit the multivariate analysis (e.g. history of psychotropic medications, current personality disorder).

**We added limitations taking up these elements at the end of the discussion:** “Besides the limited number of SRL patients enrolled in the study, we note differences at baseline between patients and controls concerning current personality disorders and past anxiolytic and hypnotic treatments. Moreover, the follow-up was not identical for all the patients since only 76% of them attended the last visit, which could have led to an underestimation of the development of axis I psychiatric disorders; however, it is important to emphasize that there were no differences between them regarding the MADRS and HAMA scores at baseline.”

V. Conclusion:
• By reading the conclusion the impression made is that the patients have no psychiatric morbidity neither currently nor in the past which is not the case. The sentence should be caveated to convey the presence of more personality disorders and more past psychotropic treatment.

**We changed the sentence accordingly:** “In conclusion, this study shows that SRL patients who were not suffering from current axis I psychiatric disorders nevertheless experienced numerous neurovegetative, somatic and psychic anxiety symptoms. Those with marked symptoms of psychic anxiety were at risk of developing ulterior psychiatric disorders.”

Reviewer #3: Dear Authors and Editors,

1. 5 patients took Antidepressant and 4 took Anxiolytics but they had no Axis I diagnosis. Authors should clearly report what antidepressants/anxiolytics they used for which reasons. Otherwise, it may be conflicting with their inclusion criteria.

**One patient with “anxiolytic” was corrected in “hypnotic”. For the others, it is the same patients who have antidepressants and anxiolytics with long-term treatments (between 2.5 and 30 years of duration). Indications for treatment were depression (n=3) and anxiety disorders (one phobia and one panic disorder). Even if some treatments do not seem to comply with the recommendations, a psychiatrist checked that the patients did not have current axis I psychiatric disorders at the time of inclusion. Lastly, it should be noted that even if current psychotropic treatment is associated with high scores of psychic and somatic anxiety, taking treatment is not associated with the occurrence of psychiatric disorders during follow-up.**

2. 12 controls were on Current psychiatrist consultation. 10 controls took Antidepressants and 14 took Anxiolytic. The control selection was inappropriate because those with skin-limited LE having current Axis 1 diagnosis were excluded from the case sample. Those with current Axis 1 diagnosis should be excluded in the control sample.

**We thank you for noticing this error and we apologize for it. Regarding the controls, they had no current psychotropic treatment; these are the past psychotropic treatments which had been mistakenly reported in "current psychotropic treatment" (see table 1)**

3. I am very surprised on the prevalence of personality disorder. 32% was so high. Authors should clearly report cluster A, B, C PD prevalence, separately. In addition, I am also very surprised at the great gap between PD diagnosis and no current Axis 1 diagnosis in the case sample.
**We have reported cluster A, B, C PD prevalence, separately in table 1. There is no significant difference between patients and controls on the different cluster PD prevalence, even if patients have a little more cluster A and C PD prevalence. With regard to PD, the prevalence that we observed in our study is close to that found in other studies using the PDQ4 with the clinical significance scale (27% Bouvard; 38% Calvo)^[4]^. A PD is relatively stable over time (DSM 5). Although patients may not have current axis I psychiatric disorders, 50% of patients have had past psychiatric disorders. If we look at all the participants, 59% of those with a PD have already had an axis I psychiatric disorder.**

4. Authors should clearly report what Medical comorbidities are.

**The medical comorbidities were as follows:**

|  | Patients, n (%) | Controls, n (%) |
| --- | --- | --- |
| Infection | 2 (5) | 1 (1) |
| Tumor | 3 (8) | 7 (9) |
| Endocrine, nutritional or metabolic diseases | 3 (8) | 6 (8) |
| Circulatory system | 8 (21) | 11 (14) |
| Digestive system | 4 (11) | 8 (11) |
| Musculoskeletal system and connective tissue | 5 (13) | 6 (8) |
| Genitourinary system | 2 (5) | 2 (3) |
| Nervous system | 2 (5) | 4 (5) |
| Respiratory system | 3 (8) | 1 (1) |
| Diseases of eyes | 2 (5) | 1 (1) |
| Diseases of the ear | 1 (3) | 0 |

**In order to avoid overburdening the table, the main comorbidities have been added at the bottom of the note in Table 1.**

5. Table 2 showed selected associations between Associated factors with symptom scores in all participants. Why did authors only report 3 factors? How about other factors? such as Current psychotropic treatment...?

**Given the number of participants, the number of variables that can be included in the multivariate model is limited and we must ensure that there is no collinearity between the variables.**

**We only reported the factors associated with the scores in the univariate analysis (see Table S1 in the supplementary data). This was already specified in "Statistical method" (**“*Multivariate analyses of factors associated with the symptom scores were performed by multiple generalized linear regression, taking the group (patients vs controls) as main covariate and selecting other covariates, based on clinically relevant factors or statistically significant factors shown in univariate analysis”****),* then in the “Results” *(****“To understand why patients had higher scores than controls and to identify the factors responsible for the differences, we performed multiple generalized linear regression using the study population (patients vs controls) and the previously identified factors (current personality disorders and past psychotropic treatment) as covariates. The results are presented in table 2.”)*.

**New results were obtained following the correction concerning “psychotropic treatments”: current psychotropic treatment is associated with higher anxiety and somatic scores (see S1 Table). We therefore added current psychotropic treatment to the multivariate model (after checking that there was no collinearity between the variables). Scores aren’t associated with current psychotropic treatment. Only one change is observed: a stronger association between being a patient and the somatic anxiety score (p=0.09).**

6. This study seemed to be a follow-up study. But, the related information was very limited in the method section. How long were those participants followed? When was the second assessment? Were controls followed?
**We agree that relying on previous papers for the description of the LuPsy cohort is not sufficient; we have therefore added a brief description in the “Participants” part of the “Method” section:** *“The patients were followed for 2 years with a visit every 6 months (5 visits in total) including both a dermatological and a psychiatric evaluation. Controls had no follow-up.”*

7. In the results, "The main factors identified were.....had higher depressive and psychic anxiety symptom scores than patients without". This analysis was done based on all participants. I supposed some were not patients.

**We thank you for noticing this error and we apologize for it. The term “patients” has been replaced by “participants” in the text.**

8. "....the other 33 underwent 4.2 assessments on average, with an average follow-up of 21.9 ± 5.9 months."...Did it mean each patient were followed with different duration and at different time? Is it a standard cohort study? Why was the follow-up timepoint so different?
**A dermatological and psychiatric evaluation was carried out every 6 +/-1 months. It is therefore possible that all the patients didn’t have exactly the same follow-up. Moreover, as indicated in the results, only 76% of patients attended the last visit.**

**Among patients who had a psychiatric disorder, follow-up was 20.4 +/- 6.9 months; among patients without a psychiatric disorder, follow-up was 22.4+/-5.6 months; there is no difference considering the standard deviation.**

**This could have led to an underestimation of the development of axis I psychiatric disorders.**

**Those lost to follow-up could be patients who have had a psychiatric disorder. However, it is important to emphasize that there were no differences regarding the MADRS and HAMA scores at baseline between the patients who attended the last visit and those who didn’t.**

**These points have been discussed in the limits.**

**“**Besides the limited number of SRL patients enrolled in the study, we note differences at baseline between patients and controls concerning current personality disorders and past anxiolytic and hypnotic treatments. Moreover, the follow-up was not identical for all the patients since only 76% of them attended the last visit, which could have led to an underestimation of the development of axis I psychiatric disorders; however, it is important to emphasize that there were no differences between them regarding the MADRS and HAMA scores at baseline.”

9. "The Fig 2B revealed that the patients who developed a psychiatric disorder during the follow-up had a distinctive baseline profile than those who did not develop such disorders."...Psychiatric disorder has many diagnoses...So, what was the definite diagnosis in such patients who developed a psychiatric disorder? Was it reasonable to compare the baseline difference between patients with or without a psychiatric disorder because a psychiatric disorder was so heterogenous?

**Psychiatric disorders that occurred during the follow-up are three depressive disorders (including one with anxiety disorder), two dysthymia, one suicide risk, two panic disorders and one social phobia.**

**Almost all these disorders are depressive or anxious disorders whose symptoms can be identified and measured on anxiety and depression scales such as MADRS and HAMA.**

**The description of the disorders has been added in the "Results" section.**

Reviewer #4: The Authors have addressed all points raised by the referee(s). The manuscript (including the tables/figures) have been revised accordingly.

1. Dowlatshahi EA, Wakkee M, Arends LR, Nijsten T. The prevalence and odds of depressive symptoms and clinical depression in psoriasis patients: a systematic review and meta-analysis. J Invest Dermatol. juin 2014;134(6):1542‑51.

   Marrie RA, Walld R, Bolton JM, Sareen J, Walker JR, Patten SB, et al. Increased incidence of psychiatric disorders in immune-mediated inflammatory disease. J Psychosom Res. 2017;101:17‑23.

   Rønnstad ATM, Halling-Overgaard AS, Hamann CR, Skov L, Egeberg A, Thyssen JP. Association of atopic dermatitis with depression, anxiety, and suicidal ideation in children and adults: A systematic review and meta-analysis. J Am Acad Dermatol. sept 2018;79(3):448-456.e30.

   Zhang L, Fu T, Yin R, Zhang Q, Shen B. Prevalence of depression and anxiety in systemic lupus erythematosus: a systematic review and meta-analysis. BMC Psychiatry. 14 2017;17(1):70.

   Jalenques I, Bourlot F, Martinez E, Pereira B, D’Incan M, Lauron S, et al. Prevalence and Odds of Anxiety Disorders and Anxiety Symptoms in Children and Adults with Psoriasis: Systematic Review and Meta-analysis. Acta Derm Venereol. 26 août 2022;102:adv00769.
2. Cuijpers P, Smit F. Subthreshold depression as a risk indicator for major depressive disorder: a systematic review of prospective studies. Acta Psychiatr Scand. mai 2004;109(5):325‑31.

   Korte KJ, Brown MAS, Schmidt NB. Anxiety sensitivity and impairment: evidence for a direct association and partial mediation by subclinical anxiety symptoms. J Affect Disord. déc 2013;151(3):875‑81.
3. Jorm AF, Griffiths KM. Population promotion of informal self-help strategies for early intervention against depression and anxiety. Psychol Med. janv 2006;36(1):3‑6.
4. M Bouvard, P Cosma. [An exploratory study of a personality disorders questionnaire]. Encephale 2008;34(5):517-25.

   Natalia Calvo 1 , Fernando Gutiérrez, Miguel Casas. Diagnostic agreement between the Personality Diagnostic Questionnaire-4+ (PDQ-4+) and its Clinical Significance Scale. Psicothema 2013;25(4):427-32.
