# Supplementary material for: Psychiatric symptomatology in skin-restricted lupus patients without axis I psychiatric disorders: A post-hoc analysis
Source: PLoS One. 2023 Mar 1;18(3):e0282079. doi: 10.1371/journal.pone.0282079 (PMC9977055; doi:10.1371/journal.pone.0282079)
Supplement: Attachment — Submitted filename: Response to reviewer.docx [file pone.0282079.s007.docx]

Reviewer #3: Dear Authors and Editors,

*1. For me, it is not very reasonable for the reply that "Indications for treatment were depression (n=3) and anxiety disorders (one phobia and one panic disorder)" and "a psychiatrist checked that the patients did not have current axis I psychiatric disorders at the time of inclusion". I suppose authors may indicate no active psychiatric episode, or remitted state of a psychiatric disorder. If no diagnosis, why did those patients take medications?*

**We understand your comment. Indeed all these patients have a history of psychiatric disorders but no active psychiatric episode. Moreover, these patients had these treatments at the time of inclusion for several years and are not always linked to a recent episode. In order to avoid any confusion, each time it was relevant to underline the absence of an active psychiatric episode, we have added in the text “no active psychiatric disorder”.**

*2. I am confused for the definition of Axis I and II diagnoses in current study. Is Axis I diagnosis based on a psychiatrist's interview? Is Axis II diagnosis based on the 99-item self-report Personality Diagnostic Questionnaire? Axis I and Axis II are clinical diagnoses. I am not sure whether authors used different modality to define those diagnoses. In addition, based on DSM 5 (as authors reported), the concept of axis diagnosis is not applied.*

**Indeed Axis I diagnosis is based on a structured psychiatrist's interview, the MINI, and axis II diagnosis is based on the 99-item self-report Personality Diagnostic Questionnaire as mentioned in the method section: “***The past and current Axis I psychiatric disorders were explored by a psychiatrist using a structured interview, the MINI 5.0.0 [25]. The 99-item self-report Personality Diagnostic Questionnaire 4+ (PDQ-4+) was used to assess personality disorders.****”*We have chosen to use the PDQ-4+ questionnaire to explore personality disorders because its use is simple and allows the doctor to check the results using a scale of clinical significance. Since the diagnosis of personality disorders is difficult and time-consuming, using this questionnaire with the scale of clinical significance is a good alternative for research.**

**The interview (MINI version 5.0.0) and the PDQ4 questionnaire are both based on the DSM IV-TR and not on the DSM5. We had mentioned the DSM5 in our article only in the introduction when we pointed out that the presence of symptoms does not always imply the presence of psychiatric disorders. Since this note is true for different versions of the DSM, we have removed the DSM5 precision to avoid confusion: “***In this inflammatory context, other patients can develop anxious and depressive symptoms that are not severe enough to meet DSM criteria for depression and anxiety disorders but which correspond to subclinical disorders***”.**

*3. "The psychiatrist also recorded scores from the Montgomery-Asberg Depression Rating Scale (MADRS) [30] and the Hamilton Anxiety Rating scale (HAMA) [31]"..Authors should report the symptom scores at baseline and during the follow-up between cases and controls.*

**The MADRS and HAMA baseline scores were only presented in Figure 1. We have added the values in Table 1.**

**As indicated in the "Participants" section, controls do not have a follow-up visit: “***The patients were followed for 2 years with a visit every 6 months (overall 5 visits) including both a dermatological and a psychiatric evaluation. Controls had no follow-up***”, only patients have multiple visits.**

**The evolution of patients' MADRS and HAMA scores during the visits has been added as supplementary data (S1 Fig):**

*4. based on figure 1, we can see several controls rated >= 10 on MADRS and even more cases rated >= 10 on MADRS. The remission criteria of MADRS is commonly defined at 10. Authors said a psychiatrist diagnosed no axis 1 diagnosis in cases and controls, which were conflicting with the rating scores.*

**Indeed, a MADRS score of less than 10 is most often retained in therapeutic trials to indicate remission of depression. However, the MADRS is not a diagnostic tool for depression that meets DSM criteria, but rather a tool to measure severity and response to treatment. We can also note that clinical studies including patients suffering from depression generally show MADRS scores ≥ 24.**

**The presence of the disorders was assessed using the Mini International Neuropsychiatric Interview (MINI). This structured diagnostic psychiatric interview has very good psychometric qualities for the detection of major depressive disorders (sensitivity between 86% and 96% and specificity between 79% and 88% with SCID-P, CIDI and expert diagnoses) with an excellent interrater reliability (kappa=1.00) (1998 Sheehan(1)).**

**The MADRS is a validated tool that has good psychometric qualities. However, its sensitivity and specificity are not 100%. Here are several examples of studies giving the specificity and sensitivity of the MADRS compared to standardized clinical interviews such as the MINI:**

|  |  |  | **MADRS** | | |
| --- | --- | --- | --- | --- | --- |
| **Study** | **Population** | **Depression diagnostic** | **cut-off** | **Sensitivity** | **Specificity** |
| 2010 Bernstein (2) | Psychiatric outpatients | MINI | 8 | 97% | 33% |
|  |  |  | 12 | 92% | 59% |
| 2012 Bunevicius (3) | Coronary artery disease | MINI | 10 | 88% | 85% |
| 2014 Kjaergaard (4) | Healthy population | SCID | 10 | 70% | 89% |

**Therefore the existence of false positives cannot be ruled out and participants who have MADRS scores greater than 10 in our study could be among them. Note that in participants with MADRS scores greater than 10, the most impacted items in the MADRS (inner tension and reduced sleep) are not the items most specifically linked to depression (sadness, suicide, pessimistic ideas, inability to feel).**

1.	Sheehan DV, Lecrubier Y, Sheehan KH, Amorim P, Janavs J, Weiller E, et al. The Mini-International Neuropsychiatric Interview (M.I.N.I.): the development and validation of a structured diagnostic psychiatric interview for DSM-IV and ICD-10. J Clin Psychiatry. 1998;59 Suppl 20:22-33;quiz 34-57.

2.	Bernstein IH, Rush AJ, Stegman D, Macleod L, Witte B, Trivedi MH. A Comparison of the QIDS-C16, QIDS-SR16, and the MADRS in an Adult Outpatient Clinical Sample. CNS Spectrums. juill 2010;15(7):458‑68.

3.	Bunevicius A, Staniute M, Brozaitiene J, Pommer AM, Pop VJM, Montgomery SA, et al. Evaluation of depressive symptoms in patients with coronary artery disease using the Montgomery Åsberg Depression Rating Scale. International Clinical Psychopharmacology. sept 2012;27(5):249.

4.	Kjærgaard M, Arfwedson Wang CE, Waterloo K, Jorde R. A study of the psychometric properties of the Beck Depression Inventory-II, the Montgomery and Åsberg Depression Rating Scale, and the Hospital Anxiety and Depression Scale in a sample from a healthy population. Scandinavian Journal of Psychology. 2014;55(1):83‑9.
